# Psychosocial and Ergonomic Conditions at Work: Influence on the Probability of a Workplace Accident

**DOI:** 10.1155/2019/2519020

**Published:** 2019-11-11

**Authors:** J. R. López-García, S. García-Herrero, J. M. Gutiérrez, M. A. Mariscal

**Affiliations:** ^1^Escuela Politécnica Superior, Universidad de Burgos, Burgos, Spain; ^2^Consejo Superior de Investigaciones CientÍficas, Instituto de Física de Cantabria, Santander, Spain

## Abstract

Today, the economic and social importance of occupational accidents is undeniable worldwide. Hence, research aimed at reducing this type of accident is considered a discipline of great interest for society in general. In this environment, working conditions play a fundamental role in the occurrence of accidents, and from their study, results can be obtained that provide information for decision-making that guarantee optimum conditions for the development of the employees' tasks. Organizing the conditions of work execution is also a task that constitutes an essential aspect for a firm's productivity, therefore, affecting their viability and results. In this work, a model is proposed for the study of different groups of working conditions and their influence on the probability of occupational accidents, in accordance with the data provided by the 7^th^ National Survey of Working Conditions (VII NSWC). The survey sampled 8892 workers active in all sectors of national production and is the last nation-wide survey administered in Spain. Bayesian networks (BNs) are used to generate a network that analyzes working conditions in all areas (27 variables have been included in addition to those corresponding to the sector and accident), and then, more specifically, the relationship that is established between ergonomic factors in the workplace, psychosocial factors of the worker, and the probability of an accident. The results are achieved through the network obtained by highlighting some of the proposed variables. The dependencies generated by the chosen variables are analyzed, and subsequently, the probability of accident for each of the productive sectors is determined. It is concluded that the ergonomic risks associated with physical strains in the workplace, together with the lack of job satisfaction on the employer's behalf, both pose a very significant increase in the probability of being involved in an occupational accident, above the other variables of study.

## 1. Introduction

The development of scientific knowledge for study of occupational accident rates, the search for causality, and subsequent data treatment focused on reducing those rates in the workplace, is the priority raised by this article. Occupational accident rates remain at worrying levels with significant socioeconomic consequences in all countries, and its decrease is the goal that all parts involved must pursue.

The competitiveness of companies as well as their success are, in turn, conditioned by this problem because the worker's productivity is affected, and therefore, production costs increase.

The current legislation in Spain requires ensuring health and safety at work as one of the guiding principles of social and economic policy, and being directly derived from is the determination of the basic body of guarantees and responsibilities which are necessary to establish an adequate level of protection for the workers' health against the risks that might arise from working conditions [1].

In Spain, the statistical data report a sharp decrease in occupational accidents over the period between 2008 and 2013; the lowest number of occupational accidents in the daytime having been registered in 2013. As from that year, the numbers of occupational accidents have increased up until 2017.

**Figure 1:**
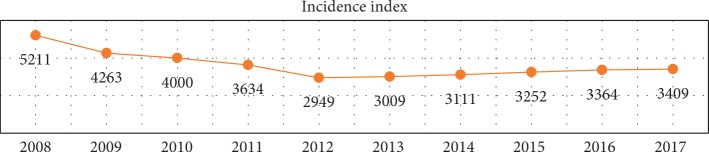
Incidence index of occupational accidents over the period 2008–2017. Source: “Anuario Estadisticas” MEYSS.

It is evident that the economic recession, from 2007 to 2013, led to a significant decrease on the number of active workers, and by observing the incidence rate (equation (1), source: INSHT), the tendency coincides, but from 2013, the trend changes and the number of accidents increases (Figure 1):

(1) $$\begin{array}{c} \text{incidence index }\left(i\;=\;\frac{\text{num}.\text{ of accidents in the working day with sick leave}\times 10^{5}}{\text{average num. of workers exposed}}\right). \end{array}$$

The present study is based on data provided by the VII National Survey of Working Conditions (VII ENCT) published in 2012 by the National Institute of Occupational Safety and Hygiene [2]. This database gathers workers' responses on their working conditions and studies all relevant aspects of workers' conditions and their relationship to safety and health. These data are obtained on the basis of a questionnaire that contemplates the different disciplines of occupational risk prevention and also provides data on the working conditions of each survey respondent in the demographic and the labour market.

The aim of this paper is to analyze the relationship between working conditions and the probability of experiencing an occupational accident, picking out individual variables from the ergonomic conditions of the employment post and the psychosocial aspects of the worker. Likewise, a search was conducted for the most significant variables, to observe their implication in the increase/decrease of the probability of an accident.

The investigation of occupational accidents is the search for factors that intervene in the genesis of accidents, seeking out causes and not guilty parties. The objective of the investigation is the prevention seeking to neutralize the risk from source or at its origin, refusing to assume that its consequences will be inevitable.

In the proposed model for the development of this research, which we will see below, various working conditions have been taken into account, covering multiple aspects related to the environment in which workers carry out their activities. 27 variables are integrated into the model to create the Bayesian network, but in order to obtain results, only those conditions related to ergonomics and psychosociology (6 variables) will be used. Next, the focus will be put on the bibliographic analysis of the variables related to these subjects and their effect on workers' health condition.

The ergonomic and psychosocial disciplines used in the present study are aspects that have been studied by different authors in relation to occupational accidents. Both ergonomics and the aspects related to psychosociology in the workplace have to be taken into account and integrated in the Health Risk Prevention Plans in the workplace [3] in order to improve the worker's health and safety conditions.

The recent literature review carried out by Hanvold et al. [4] shows that ergonomic factors along with psychosocial factors, such as the worker's autonomy and a safe environment, are associated with an increased risk of injury for young workers.

### 1.1. Ergonomic Factors

Ergonomic factors are of great importance, not only in relation to the worker's own health and safety but also in other aspects associated with production. Ergonomic requirements are an essential element in the quality of the work that is performed according to the study developed by Górny [5]. It is also stressed that ergonomic variables have to be considered in the development of quality management plans, in order to guarantee acceptable conditions that will permit the completion of the tasks to the highest quality [6].

Sustained physical work can be the cause of bodily injury to workers, which in turn entails enormous losses to the industry in terms of money, time, and productivity. Several safety and health organizations have proposed rules and regulations that limit workers' efforts in order to mitigate possible bodily injuries [7]. However, physical efforts continue to cause serious damage to workers' health and, as a result, managers' efforts should be directed towards a more ergonomic and safe working environment [8].

In an analysis of the relation between ergonomics—accidentally, in the study of Saari et al. [9]—the experience of the worker in the workplace, (monotonous) repetition, and mobility in the workplace, were proposed as the variables most closely linked to the occurrence of accidents.

The study of ergonomically unacceptable working conditions completed by Kilbom and Broberg [10] concluded that women perform repetitive and monotonous tasks in manufacturing industry with greater frequency than men, which is apparent in injuries that principally affect the neck, the arm, and the shoulder.

One of the causes of higher accident rates, in developed countries, is linked to the increasing installation of machinery. Lack of protection on machines, poor maintenance, and inexperienced workers was reported as the principal factors in the occurrence of occupational accidents [11].

In the construction sector, the problems arising from personal equipment associated with the job, faulty equipment, and auxiliary aids, the suitability of the materials that are employed, and shortcomings in safety management have been linked to the occurrence of accidents [12–14].

The comparison of the ergonomics of positions of employment and their problems in different countries was examined by Bhattacherjee et al. [15] through a study of the working population of the mining sectors both in India and in France. In the Asian sample, most of the injuries took place due to handling of tools, materials, and machinery, as well as due to the problems linked to environmental variables. In France, the injuries appeared to be related with biomechanics, an aspect that is related with the age of the workers and their physical state. On workers in the United States, Bobick et al. [16] considered that musculoskeletal injuries were the most common and they concluded with the need to design the employment position, taking into account the physical consequences for the worker.

Related with the design of the post or work station, adopting inappropriate postures while working and the lack of safety preparation were shown to be the principal causes of accidents [17].

The association between workload and performance of public transportation drivers is analyzed in the study by Useche et al. [18], which supports a negative effect of workload on professional performance in relation to aspects such as traffic accidents and penalties.

### 1.2. Psychosocial Factors

The associations between psychosocial factors and sick leave, both due to illness and due to accident, have been examined through aspects related with the work environment, management quality in the firm, and the conciliation of work and family life, among others.

A working environment that implies placing high demands on people and that provides little supervision over the completion of tasks will limit self-esteem and will, therefore, provoke a stressful experience with adverse long-term health-related consequences [19]. Fatigue associated with a high workload, jobs with high psychological demands, and personal conflicts with fellow workers are risk factors to control in view of possible mishaps [20].

An aspect such as the autonomy when deciding the work schedule was manifested as an attenuation of the frequency of injuries. It also places work satisfaction and work-life balance as a mitigating factor for possible occupational accidents [21].

Managerial leadership appears as an element to take into account in worker pathologies of a psychosocial type [22]. In their article, Hinkka et al. [23] attributed a reduction in the risk of accidents, resulting in a sick leave to positive encouragement from managers, to a good working climate, and to recognition at work. Along the same lines, Giorgi et al. [24] considered that managers have to develop mechanisms for the detection and the supervision of the psychological health of their workers and specifically the stress that they undergo for the improvement of the conditions that cause it. In the study carried out by [25], the results support when risk management takes into account the psychosocial aspect of workers. There is also evidence that there has been collaboration with workers to demonstrate that they are valued, among other factors.

The emotional conditions and the social relations of people are aspects that have some importance in the occurrence of accidents [26]. The study conducted by Kirschenbaum et al. [27] concluded that there was a considerable probability of injuries when observing the type of employment position, the level of personal income, involvement in dangerous jobs, emotional concern, and poor living quarters. The existing relationship between work dissatisfaction and its consequences on worker's health is demonstrated when a low satisfaction increases the levels of risk of injury. This correlation suggests the need for interventional methods to detect these kinds of situations [28].

The existence of bullying at work is another aspect that deteriorates the occupational satisfaction of bullied workers, with harmful consequences for the health of the worker [29, 30]. The state of satisfaction with the position of employment is revealed as a factor that diminishes the risk of involvement in an occupational accident, above all in jobs with greater specialist demands that have a direct effect on workplace injuries [31].

The influence of psychosocial conditions analyzed by gender yields as a consequence of very differentiated situations [32]. In small and medium firms, stress and workload generate high indices of association with accidents among men; nevertheless, scarce few relations with workmates and family are demonstrably the most significant factors among women.

The association between occupational risks and the perceived mental health of individuals is analyzed using the 5th European Working Conditions Survey (V EWCS). It is proven and stressed the need to act to improve the mental well-being of workers to minimise their exposure to states that could pose a risk to their health while at work [33].

Occupational stress is without a doubt one of the most extensive psychosocial consequences among the working population of developed countries and is, therefore, one aspect of prevention that is currently the subject of exhaustive studies [34].

The study of the exposure of the salaried working population in Spain to psychosocial risks between 2004 and 2005 highlighted poor leadership quality, emotional psychological demands, and possibilities of professional development as the most unfavourable for employee health [35].

Recently, the study conducted by Coupaud [36] on the analysis of psychosocial conditions associated with the health of workers in Europe over the period 2000–2015 showed a close relation between interpersonal relations and worsening health. Social support is important, both from superiors and peers. The risk of physical injuries increases significantly (3.5 times) among individuals with risk exposures and under the support of their supervisor, as compared to peers with low exposure and direct support [37]. According to the research work done by Morag and Luria [38], the development of group participation systems for risk prevention is positive.

## 2. Data

The data used in the present study were provided by the 7th National Survey of Working Conditions (VII NSWC), a survey completed in Spain by the National Institute of Safety and Hygiene at Work (INSHT), an organism reporting to the Ministry of Employment and Social Security. It was administered between 2011 and 2012, following the lines marked out by earlier editions, with the objective of contributing updated information on the working conditions of the different sectors into which the working population of Spain is grouped.

The survey covered a sample of 8892 workers (Table 1) interviewed at home through a questionnaire with a total of 62 questions [2]. The scope to which the survey was administered consisted of individuals aged over 16, in full-time employment, in all economic activities. The sample was distributed in accordance with the number of active employees according to the Active Population Survey average 2009 (EPA 2009) adapted to an initially foreseen sample of 9000 workers. Weighted coefficients were applied to adjust the sample to the situation described in the EPA 2009, which attempted to adjust some of the study groups to those figures.

The response to question Q-52 “Have you experienced an occupational accident over the past two years?” was considered essential to conduct the study. On the basis of the survey, 12 respondents gave no answer to this question, which explains the reduction in sample size from 8892 to 8880 workers. Neither were the abovementioned weighted coefficients applied.

The 27 chosen variables from the national survey were divided into six large groups that, together with the variables accident and sector, are presented in Table 2. The frequency of the responses from the workers was also included in accordance with the categories of each question under consideration. These categories were defined by the authors is such a way as to simplify the different responses that the survey respondents could select.

It should be noted that the questions raised in the groups relating to ergonomics and psychosociology implied multiple questions. The responses were therefore grouped on the basis of the coefficient “alpha's Cronbach” [39, 40]. This coefficient is considered a measure of reliability that enabled us to group the responses to different interrelated questions under a single variable, by taking into account the different responses reflected in the same question. The values of this indicator can be anywhere between 0 and 1, with values between 0.70/1.00 reflecting acceptable/excellent reliability.

## 3. Methodology

The methodology based on probabilistic networks, known more specifically as Bayesian networks (BN), was chosen to conduct this study. They are described as “*combining graphs and probability functions to define probabilistic models in an efficient manner that contain the desired relations of dependency for a problem and that are computationally processed*” [41]. The BN infers the joint probability function (JPF) from the data, based on the dependency relations defined in the graph. This function employs algorithms to relate the probabilities between each other, some of which are based on the Bayes equation [42]. A characteristic of the BN and the “machine learning” methods is the possibility of implementing machine algorithms [43].

The sampling data, explanatory variables, and objective were all included in the knowledge base because there was no other way of inferring its probability, given factor (variable)-based evidence. Evidence is a defined value that is labelled a variable at a given point in time. This process is known as inference or probabilistic reasoning and is used to quantify the uncertainty of different problem-related variables as the evidence is introduced [41].

MATLAB software was used to generate the network, through a specially designed code with the MATLAB toolbox, known as METEOLAB (Meteorological Machine Learning Toolbox for MATLAB), designed by the Santander Meteorology Group (Santander.Met.Group).

The network that was generated to obtain the results was characterized by the definition of 29 variables related with the working conditions, including the variable V1 (accident) established as an objective variable for the purposes of this study. In this way and through the introduction of evidences linked to other variables (different categories are defined in each one), the probabilities of an occupational accident are computed as functions of the evidence/s that is/are introduced.

## 4. Model and Variables

### 4.1. Proposed Model

The Bayesian network was obtained by selecting the 29 selected variables, drawn from the responses to the survey questionnaire. They were likewise grouped according to six criteria:

- Demographics: group data related with the geographic situation and personal data of the worker
- Labour market: data related to the type of contract and experience, including variables on the characteristics of the post
- Occupational safety: safety conditions of the post
- Occupational hygiene: external conditioners that are present in their post
- Ergonomics of the post: physical and emotional effort demanded for the completion of the work
- Work-related psychosociology: conditioners related to the satisfaction of the worker with regard to the position of employment and the social surroundings

**Figure 2:**
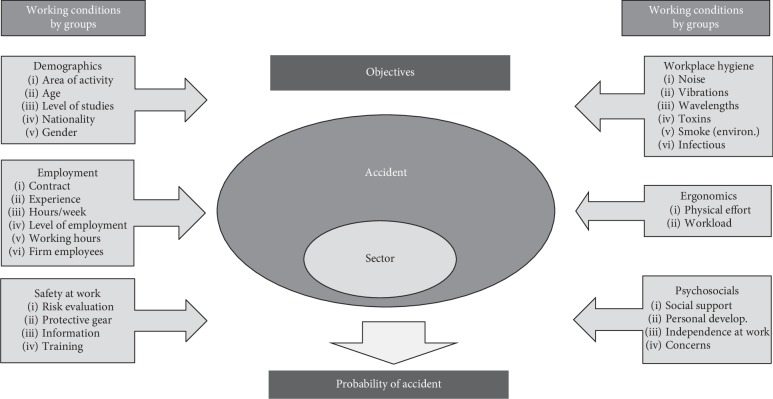
Proposed model. Source: authors.

In this way, a model is built to generate the network (Figure 2), and subsequently, evidencing certain variables in accordance with the model, the probability of an occupational accident is obtained in relation to the variables under consideration. The results related to the variables that are considered alongside the occupational accident rate across the sector of activity will also be obtained.

### 4.2. Study Variables

**Figure 3:**
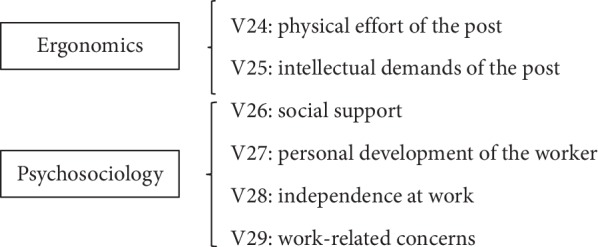


As it was earlier indicated, the purpose of this work is to attempt to obtain the relation between accident probabilities (V1) and the variables of both the ergonomics and the psychosociology groups (Figure 3).

In what follows, we will define the 6 variables corresponding to the two groups of study and all of them will categorized on a 5-point Likert-type scale.

#### 4.2.1. V24: Physical Effort of the Post

This variable summarizes the multiple responses to Q-28 of the questionnaire (Table 3). Many aspects of the design of the position of employment corresponds to the question “*how frequently are you exposed to …*”. The following situations were proposed.

In view of the frequencies obtained after grouping the variable values, it was decided not to use the response “*always*,” given the scarce few times it was used and the results of which would suppose a low statistical significance (Table 2).

#### 4.2.2. V25: Workload

In a similar way to the earlier variable, Q-30 has nine response options (Table 4), as optional responses to the question “*how frequently do you have to …*”. The following situations were proposed.

#### 4.2.3. V26: Social Support

Q-31 of the survey attempts to reflect the worker-related social environment with workmates and managers through a series of questions under the same heading, “*how frequently do you …*?”, and offers a series of options that can be divided into two parts, on the one hand, making reference to the support of managers and workmates (Table 5), and on the other hand, to aspects related with the personal development of the worker (Table 6).

#### 4.2.4. V27: Personal Development

The second block of questions included in Q-31, corresponding to the personal development of the worker (Table 6), similar to the concept of “*Empowerment*” in the Anglo-Saxon world, set out the following situations.

#### 4.2.5. V28: Autonomy

Among the circumstances that surround the worker at his post are the opportunity of changing the development of the working activity and thereby collaboration in the decisions. Q-32 proposes four possibilities (Table 7).

#### 4.2.6. V29: Concerns

There are different aspects of the study that can generate concern among workers, which is why knowledge of those aspects for their analysis is appropriate in this piece of work. Q-55 of the survey has a direct impact on those situations that might be a cause of concern for the worker (Table 8). It sets out 18 causes that might generate problems.

## 5. Bayesian Network

### 5.1. BN Graph

**Figure 4:**
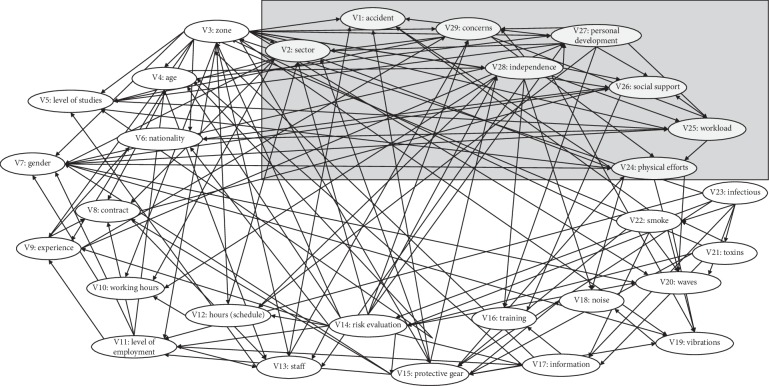
Graph of the Bayesian network. Source: authors.

Having introduced the totality of the variables selected in the survey and having generated the resulting network, we can observe variable V1 (accident) in the upper zone of the graph and its relations with different network variables and the rest of the variables placed in an anticlockwise direction (Figure 4).

**Figure 5:**
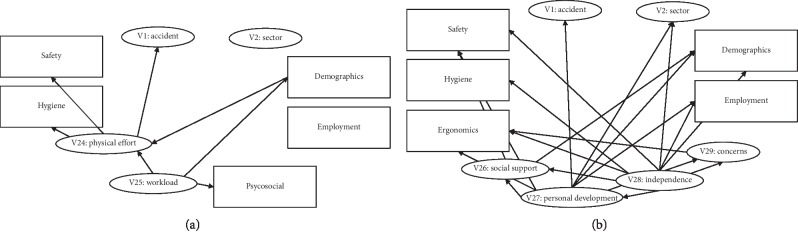
Graph of the Bayesian network. Source: authors.

Among the variables under consideration for this study, only two of them are “directly linked” in the graph of the Bayesian network with variable V1 (accident), which gives them a stronger tie of dependency (Figures 4 and 5):

  V24: physical effort of the post
  V27: personal development (empowerment)

### 5.2. BN Validation

The validation of the Bayesian network was done through the measurement of the area under the ROC curve (AUC—area under the ROC curve). The analysis of receiver operating characteristics (ROC) is displayed on a graph, in other words, in the bidimensional representation of the points resulting from the application of two measurements. In the sensitivity (S) ROC graphs, the true positives are represented on the *Y*-axis and 1-specificity (1-S) on the *X*-axis, in other words, the ratio of false positives. A ROC graph represents the relative scale between benefits (real positives) and costs (false positives), thereby modifying the decision-making threshold [44].

The calculation of ROC is a widely used index that summarizes the behaviour and the precision of the classifier [45], thereby validating the data that were collected. The methodology evaluated the capacity that these variables have in predicting the occurrence of an accident in the workplace, in such a way that the figures attached to accident probability are reliable.

The AUC measurement was done through a cross validation for which the AUC values were obtained for the prediction of each subset under consideration and for the prediction of the whole sample by merging the predictions of each subset [46].

MATLAB software was used to perform the validation task of the network that was generated, through a code programmed for that purpose by the Santander Meteorology Group [47]. This code was implemented to carry out the evaluation of the network by applying a *k*-fold cross validation. In this method, the sample is divided into ten equal parts (*k-fold* = 10) in such a way that one of those 10 parts behaves as a validation set, leaving the rest (90%) as a training set, and this process is repeated with each partition of the initial set. In this way, given that the collected sample is made up of 8880 items, the ROC measurement is done in ten iterations, considering the training sample of 7992 items and its validation with 888 items.

Having completed the validation process, an average of 0.816 was obtained for the prediction that an accident might take place using the proposed network.

## 6. Results

A study on accident probability has been completed with evidence on the six variables selected from the 7th NSWC database, as well as on the sector of activity.

At first, the probability of each variable independently showing the accident probabilities by the categories of each variable is obtained. The analysis of the highest probabilities obtained from the variables considered through the production sector will also be carried out.

Then, two variables that are in a direct relation of dependency with the variable V1 (accident) will be examined, followed by a breakdown of the results by sector of activity.

### 6.1. Sensitivity Analysis of a Variable

The results of this sensitivity analysis are shown in Table 9. The probability of a workplace accident was “*a priori*” around **7.38%**, a result generated by the network formed of 27 independent variables, the accident probability, and the production sector. Applying the methodology described on the study variables (ergonomic and psychosocial), we obtain results of accident probability in each one of its categories.

The results obtained by the ergonomic variables show that, in the physical demands variable (V24), the range of probability values was somewhere between 16.59% of respondents who stated feeling this factor “*often*”, as opposed to 3.48% who “*never*” support these situations.

However, workload (V_25) was not shown to have a high influence on the probability of involvement in an accident, fluctuating between 7.74% and 6.88%, and even showing values contrary to those initially foreseen.

With regard to the psychosocial variables, their values are not especially high for an increased probability of an accident. However, the network indicated an important relation of dependency on variable V27 (personal development) with variable V1 (accident). The values fluctuated between 8.69% in the case of it happening “*rarely*”, as opposed to 6.74% in the situation “*always*”.

The support of managers and workmates in no way implied substantial variations in the probability of an accident, moving within intervals of +0.55 and −0.80 with regard to the initial probability.

The worker's independence variable (V28) shows an increase in probability, in the case of not having such possibilities, up to 8.72%, and on the contrary (“*always*”) around 6.46%.

It is also notable that the concerns with regard to the development of the work (V29) increase the probability of accident by up to 9.77% in the case of the workers who consider themselves “*quite*” concerned and falling to 6.34% in the situation of “*not at all*” concerned.

Once the results of the variables have been obtained individually, their impact on the different production sectors is shown in Table 10. The physical demands of work range from an “*a priori*” increase in probability of 2.79% in the agricultural sector to 9.47% in industry.

Labour demands generate small increases in accident probability, between 0.23% and 0.55% in services and industry sectors, respectively.

Social support in both the agricultural and the industrial sectors represents the highest probability increase with 0.80% and 0.85%. In construction, however, it is only 0.30%.

The obtained results, taking into account the worker's own personal development, means that, in the industrial sector, the probability increases by 2.29% and the lowest percentage or smallest increase occurs in the service sector with only 0.89%. Autonomy at work gives similar values in a range between 1.99% and 0.86% in industry and services, respectively.

Finally, work-related concerns show a higher increase in the probability in the construction sector with 3.01% and a smaller growth in services with 2.03%.

### 6.2. Sensitivity Analysis of Two Variables/Sector

As mentioned above, there are two variables (V24–V27) that show a direct relationship with the target variable (V1) through the network and by performing a sensitivity analysis of these two variables, and the values reflected in Table 11 are obtained. In order to obtain results, the two extreme situations (always-never) of variable V27 (personal development) of the worker have been proposed in order to be able to observe the degree to which it influences, as a mediating factor, the increase or decrease in the probability generated by the physical demands of the work.

Analysing those same factors filtered by the sector of activity of the workers in the survey yields, the values are shown in Table 11.

In those cases for which it was possible to perform a calculation, it can be seen that the probabilities of an occupational accident on the basis of two pieces of evidence reflect interesting results. The influence on this probability of variable V27 (personal development) (empowerment) assumes enormous importance when included in the situation of high physical effort demanded from the worker, differentiating between the situation of job satisfaction and its absence (*never*). The probability of an accident was in any case high; in the case of never having had the possibility of personal development in the workplace that same probability rose to 49.36%.

Likewise, the probabilities in the other cases reflect significant differences when the situation of job satisfaction is or is not present, except in the case of rarely making physical effort.

By production sectors, the services sector was the one with the highest probability of workplace accidents in jobs with high physical effort and the impossibility of personal development (satisfaction) of the worker, reaching 64.28%.

The other sectors made it clear that the highest probabilities were found within an intermediate degree of physical effort together with the inexistence of personal satisfaction in the workplace, varying between 18.16% in industry to 30.40% in construction work.

## 7. Discussion

Based on the studied variables, the results elucidate interesting conclusions. The BN graph indicates that the two variables directly related with V1 (accident) are V24 (physical effort) and V27 (personal development).

Considering 7.38% as an initial accident probability, the values that marked out the different variables can be observed to occupy tight margins, except for posts with high physical effort (+9.21%). This variable shows a clear relationship of dependence with the variable V1 (accident). It should be noted that the category “*always*” has not been taken into account in the results due to the low number of cases of this type.

Musculoskeletal injuries generate an enormous number of days off sick and the onset of professional illnesses that can increase the possibility of accident [7]. If to those we add that age has a negative influence on the physical capabilities of the worker, then the probabilities of an accident increase.

However, the demands of the work (V_25) do not show to have a high influence, showing values contrary to what was initially predicted. Analysing a single evidence, the probabilities obtained by ergonomics variables show that, although it seems that the demands of the work in relation to the times of completion of the work, tight deadlines, etc. are factors that we assume to be of importance in the appearance of accidents, the data extracted indicate that they do not have as much influence as physical efforts, the adoption of uncomfortable postures, repetition of movements, etc.

Regarding the psychosocial factors, the different categories of these variables generate no great differences with respect to the initial probability, although it was evident that the most unfavourable situations for the worker caused the highest accident-related probabilities: impossibility of self-development, no independence, low support, and deep concerns.

The analysis by sector indicates that the physical demands of the job clearly mark the maximum probability of accident with increases of 9.47% in the industry. This sector is the one that shows the greatest increases in all the variables studied above the rest.

When the two variables that the network directly relates with the target variable are examined, the importance of the psychosocial aspects in the increase/reduction of the probability of workplace accidents is highlighted. The intervention of supervisors and their leadership is an aspect which makes a big impact in the appearance of these risks and above all in their detection in order to minimise the effects on the worker's health [48].

The similarity of high/medium physical effort and a low level of personal development raise the probabilities to levels that warn the need to treat those aspects with special care. In the same way, the observation is worth making that high levels of satisfaction among workers in the workplace imply significant falls in the probable occurrence of accidents, above all in the service sectors [49]. This trend is repeated to a lesser extent in the agrarian and the construction sectors.

## 8. Conclusions

In first place, the importance should be highlighted of the data collected by National Survey on Working Conditions (NSWC), organized by the INSHT in Spain, for the investigation of aspects related to the prevention of occupational accidents. Reflecting the characteristics of the employment panorama in each period is of great interest for scientific studies in different branches related with the reality of the job market and its links with economic periods.

Likewise, the application of Bayesian networks widely used in various current areas of research (medicine, ecology, traffic, safety, and prevention), demonstrates its great effectiveness at the computation of probabilities that are conditional upon an event. In the case of the present study, these events are accidents, and the BN network allows us to analyze changes in the probability of such an event happening even due to other factors.

In the field of ergonomics, the physical demands of work are shown as the most determining variable in the increase in accident probability above the rest with a probability that reaches 16.59% and represents an increase of 9.21% over the initial one. It is true that the production processes and manual tasks have been focused on better welfare of the worker, but these results show that there is still room for improvement. A broader and more specialized study on the development of aspects related with physical effort is necessary, so that effort is minimized and in consequence, any health risks will be kept as low as possible. This aspect is particularly evident in the industrial sector, which generates the highest probability of suffering an accident at work. This particular sector needs to be further studied in order to promote measures to reduce workers' exposure to this type of risk.

In the field of psychosociology, the possibility of the worker doing what that worker is best at doing, being able to put into practice one's own ideas, and having the satisfaction of making something useful are revealed as conditioners that are reflected in the probability of the occurrence of an accident. In those situations where the worker is ignored, the probability increases by up to 8.69%. Evidently, this result has a lot to do with the personal satisfaction of the individual and the growth of that individual as a person, aspects which the current business leadership typologies would do well to take into account.

However, when high physical effort and low worker empowerment are added into the equation, the probability of accident rises sharply and action on these matters should be taken on various fronts. The inclusion, in business protocols, of techniques for the detection of psychosocial problems would also be recommended. Likewise, as previously indicated, the quality of management leadership is clearly needed to achieve sufficient motivation and the satisfaction of the worker in the workplace.

The implementation of measures for the knowledge of the satisfaction of workers in their job and providing mechanisms to raise the morale of staff with respect to their work is considered one of the measures to be applied in existing companies, especially in the industrial sector.

As in every other research paper, there are limitations that affect the current one as well. In this case, the data are obtained from a survey of workers. The data on reported accidents are provided by employees without being reflected in accident reports processed (sick leave) by the administration. This extreme results in a lack of data on severity, duration of sick leave, etc.

The population sample in the survey reflects the productive panorama of the country at that time, but the evolution of economies and labour markets means that it is in constant change. It is advisable to carry out subsequent studies adopting these changes. Accordingly, in the future, new studies are expected to be carried out by analysing subsequent surveys and comparing the results in order to record the evolution of working conditions and their effects on workers' health.

## Figures and Tables

**Table 1:** Sector of activity and number of workers surveyed. Source: data VII NSWC.

		Survey respondents	Percentage (%)
Sector	Agrarian	457	5.1
	Industry	1448	16.3
	Construction	599	6.7
	Services	6388	71.8
	Total	**8892**	**100.0**

**Table 2:** Variables considered for the Bayesian network. Source: data of 7th NSWC.

Group	Variables	Categories					Observation	*α*-Cronbach
Accident	V1	Yes 686	No 8194					

Sector	V2	Agrarian 456	Industry 1446	Construction 596	Services 6382			

Demographics	V3: geographical zone	North 2313	Mediterranean 2950	Centre 1753	South 1864			
	V4: age	≤34 years 2201	34 < *x* ≤ 44 2909	44 < *x* ≤ 54 2497	>54 years 1261	DK/NA 12		
	V5: educational studies	Primary 3158	Further ed. 3203	Higher ed. 2489	DK/NA 30			
	V6: nationality	Spanish 8059	Other 813	DK/NA 8				
	V7: gender	Man 4753	Woman 4127					

Employment	V8: contract	Permanent 5285	Temporary 1858	Others/self-emp. 1733	DK/NA 4			
	V9: experience	≤2 years 1764	2 < *x* ≤ 6 2568	6 < *x* ≤ 12 2118	>12 years 2406	DK/NA 24		
	V10: hours a week	≤38 h/wk. 2919	38 < *x* ≤ 40 3961	>40 h/wk. 1906	DK/NA 94			
	V11: level of employment	Employee 6311	Management 1142	Director 1395	DK/NA 32			
	V12: hours	Flexi time 3468	Full time 3161	Shifts 2017	Others 221	DK/NA 13		
	V13: staff	Micro <10 emp. 3935	SME 10–250 3693	Large >250 emp. 1252				

Safety	V14: risk evaluation	Yes 2993	No 3971	DK/NA 1916				
	V15: protective gear	Yes 2902	No 5204	DK/NA 774				
	V16: information	Yes 7653	No 1118	DK/NA 109				
	V17: training	Yes 5164	No 3613	DK/NA 103				

Hygiene	V18: noise	Yes 3164	No 5657	DK/NA 59				
	V19: vibrations	Yes 1249	No 7601	DK/NA 30				
	V20: haz. rays/waves	Yes 746	No 8099	DK/NA 35				
	V21: toxins	Yes 1347	No 7510	DK/NA 23				
	V22: smoke (environ.)	Yes 1465	No 7388	DK/NA 27				
	V23: infection	Yes 649	No 8148	DK/NA 83				

Ergonomics	V24: physical effort	Always 24	Often 262	At times 1767	Rarely 4527	Never 2300	Multiple choice	0.764
	V25: workload	Always 403	Often 2667	At times 3794	Rarely 1855	Never 161	Multiple choice	0.70

Psychosocial	V26: social support	Always 3704	Often 1615	At times 1598	Rarely 660	Never 1303	Multiple choice	0.77
	V27: personal development	Always 3021	Often 2996	At times 1852	Rarely 779	Never 232	Multiple choice	0.798
	V28: independence at work	Always 1952	Often 1671	At times 2167	Rarely 1652	Never 1438	Multiple choice	0.902
	V29: concerns	Always 2882	Often 4092	At times 1506	Rarely 369	Never 31	Multiple choice	0.902

DK/NA = do not know/no answer; SME = small and medium enterprise; Haz = hazardous.

**Table 3:** Options Q-28 7th NSWC.

1	Adopting painful and tiring postures (any part of the body: shoulder, head, arms, hands, etc.)
2	Standing up without walking
3	Seated without standing up
4	Lifting or moving heavy weights
5	Lifting or moving people
6	Applying significant force
7	Repeating the same movements of hands and/or arms
8	Little space available to work comfortably
9	Having to reach out for tools, work-related items, or objects placed at very high or very low levels or that means stretching out an arm
10	Inappropriate lighting for the job that is done (scarce, excessive, with irritating reflections, etc.)
11	Working on unstable or irregular surfaces

**Table 4:** Options Q-30 7th NSWC.

1	Maintain a high or very high level of attention
2	Work very quickly
3	Work to strict and very short deadlines
4	Attend to various tasks at the same time
5	Deal directly with people outside your firm: clients, passengers, students, patients, etc
6	Complete complex, complicated, and difficult tasks
7	Complete monotonous tasks
8	Work with computers: PC, networked computers, central computers, etc
9	Use internet/e-mail for professional purposes

**Table 5:** Options Q-31 7th NSWC.

1	Can obtain help from workmates, if requested
2	Can obtain help from directors/managers, if requested

**Table 6:** Options Q-31 7th NSWC.

1	Opportunities at work to do what you know how to do best
2	Can put into practice ones' own ideas at work
3	The sensation of doing a useful job
4	Can learn new things

**Table 7:** Options Q-32 7th NSWC.

1	The order of the tasks
2	The working method
3	The pace of work
4	The distribution and/or duration of pauses in the work

**Table 8:** Options Q-55 7th NSWC.

1	Independence at work
2	The pace of work
3	The working hours
4	The difficulty or complexity of the tasks
5	Monotony
6	The quantity of work
7	Relations with workmates
8	Relations with managers
9	Relations with other people outside the firm: clients, passengers, students, patients, etc
10	The attitudes that should be adopted
11	The physical effort that has to be made
12	The noise levels in the workplace
13	Lighting of the workplace
14	Temperature and humidity in the workplace
15	Manipulation or intake of harmful or toxic substances
16	Risk of accident
17	Risk of illness
18	Risk of redundancy

**Table 9:** Sensitivity analysis of a variable. Source: authors.

Variable	Label	Categories	% ACC	% VAR
V24	Physical effort	2—often	16.59	9.21
		3—sometimes	11.86	4.48
		4—not often	7.32	−0.06
		5—never	3.48	−3.90

V25	Workload	1—always	6.88	−0.50
		2—often	6.73	−0.65
		3—sometimes	7.74	0.36
		4—rarely	7.68	0.30
		5—never	7.02	−0.36

V26	Social support	1—always	7.36	−0.02
		2—often	7.67	0.29
		3—sometimes	7.51	0.13
		4—rarely	7.93	0.55
		5—never	6.58	−0.80

V27	Personal development (empowerment)	1—always	6.74	−0.64
		2—often	7.40	0.02
		3—sometimes	8.02	0.64
		4—rarely	8.69	1.31
		5—never	8.04	0.66

V28	Independence	1—always	6.46	−0.92
		2—often	6.64	−0.74
		3—sometimes	7.49	0.11
		4—rarely	7.97	0.59
		5—never	8.72	1.34

V29	Work-related concerns	1—none	6.34	−1.04
		2—a little	7.49	0.11
		3—regular	8.75	1.37
		4—quite a lot	9.77	2.39
		5—a great deal	7.73	0.35

**Table 10:** Sensitivity analysis of a variable and sector. Source: authors.

Variables		Category			Sector			
				Total (%)	Agrarian (%)	Industry (%)	Construction (%)	Services (%)
				7.38^*∗*^	9.52^*∗*^	9.86^*∗*^	9.98^*∗*^	6.55^*∗*^
V24	Physical effort	2	Often	16.59	12.31	19.33	17.08	15.29
V25	Workload	3	Sometimes	7.74	9.92	10.41	10.33	6.78
V26	Social support	4	Rarely	7.93	10.32	10.71	10.28	6.90
V27	Personal development (empowerment)	4	Rarely	8.69	10.82	12.15	11.50	7.44
V28	Independence	5	Never	8.72	11.49	11.85	11.62	7.41
V29	Work-related concerns	4	Quite a lot	9.77	12.07	12.76	12.99	8.58

^*∗*^“*A priori*” total and by sector accident probability values.

**Table 11:** Sensitivity analysis two variables and sector. Source: authors.

Variables			Sector			
V24 (physical effort)	V27 (personal deviation)	Total (%)	Agrarian (%)	Industry (%)	Construction (%)	Services (%)
Often	Always	12.23	14.44	12.52	11.71	12.00
	Never	49.36	—	—	—	64.28

Sometimes	Always	10.72	5.57	10.83	8.68	11.39
	Never	14.88	28.72	18.16	30.40	10.40

Rarely	Always	7.55	10.94	9.01	9.76	7.00
	Never	6.50	6.03	9.57	—	5.99

Never	Always	2.73	2.13	3.84	3.56	2.55
	Never	5.05	14.02	5.65	24.06	4.10

## Data Availability

The documentation and data used in this study (7th NSWC), previous form, can be found available on the website of the Spanish National Institute for Occupational Safety and Health at Work (INSHT) at http://encuestasnacionales.oect.es/. At the same time, through this URL you can consult online the data related to the survey and its questionnaire.
